# Evaluation of subclinical left ventricular systolic dysfunction in obese patients by global myocardial work

**DOI:** 10.1186/s13098-023-01230-7

**Published:** 2023-12-06

**Authors:** Jun Huang, Guang-an Li, Jing Wang, Yu-wen Jiao, Zhi-feng Qian, Li Fan, Li-ming Tang

**Affiliations:** 1 Department of Echocardiography, The Affiliated Changzhou Second People’s Hospital with Nanjing Medical University, Changzhou, 213003 China; 2 Department of Weight Loss Metabolic Surgery, The Affiliated Changzhou Second People’s Hospital with Nanjing Medical University, Changzhou, 213003 China

**Keywords:** Left ventricular systolic dysfunction, Obesity, Myocardial work

## Abstract

**Objective:**

To evaluate subclinical LV systolic dysfunction in obese patients by global myocardial work (MW).

**Methods:**

A total of 589 obese patients and 100 normal controls were enrolled in the study. The global longitudinal strain (GLS), global work index (GWI), global constructive work (GCW), global wasted work (GWW) and global work efficiency (GWE) were generated by a noninvasive pressure-strain loop (PSL) in apical 3-, 4- and 2-chamber views acquired by two-dimensional echocardiography. All obese patients were divided into three groups: class I obesity (mild) 30–35 kg/m^2^, class II obesity (moderate) 35–40 kg/m^2^ and class III obesity (severe) > 40 kg/m^2^. These values were compared among the three groups. The independent influencing factors of subclinical LV systolic dysfunction in obese patients were explored by constructing a multiple regression model. ROC analysis was performed to determine the performance of MW to detect subclinical LV systolic dysfunction in obese patients.

**Results:**

The absolute value of GLS in obese patients was significantly lower than that in normal controls (*P* < 0.001). The values of GWI, GCW, GWE and GCW/GWW in obese patients were significantly lower than those in normal controls (*P* < 0.05), while GWW was significantly larger than that in normal controls (*P* < 0.001). Subgroup analysis and trend analysis showed that the values of GWI, GCW, GWE and GCW/GWW in severe obese patients were lower than those in moderate obese patients and lower than those in mild obese patients (*P* < 0.01), while GWW in severe obese patients was larger than that in moderate obese patients and larger than that in mild obese patients (*P* < 0.05). Female sex, BMI and SBP were independent influencing factors of impaired GWI (*β* = 0.15, *P* < 0.001) (*β*=-0.18, *P* < 0.001) (*β* = 0.50, *P* < 0.001) and GCW (*β* = 0.17, *P* < 0.001) (*β*=-0.19, *P* < 0.001) (*β* = 0.57, *P* < 0.001). ROC analysis showed that the AUC of the combined global MW was significantly higher than the AUCs of the individual indices (*P* < 0.05).

**Conclusion:**

In this study, we conclude that subclinical LV systolic dysfunction was detected by the novel global MW technique in obese patients. Elevated BMI in obese patients results in an increased risk of subclinical LV systolic dysfunction, although the LVEF is normal. Controlling BMI in obese patients may reduce the impairment to the LV myocardial systolic function. Global MW is a novel and reproducible technique that can be well applied in the clinical evaluation of subclinical LV systolic dysfunction.

## Introduction

Obesity, with its high prevalence, is associated with various diseases, such as type 2 diabetes mellitus (T2DM), hypertension, obstructive sleep apnoea syndrome (OSAS), metabolic syndrome, and cardiovascular diseases, such as coronary artery disease (CAD) [1]. Obesity has been defined as excessive fat body mass compared to lean body mass [2]. Currently, obesity is classified based on body mass index (BMI), and patients with BMI > 30 kg/m^2^ are considered obese. According to the World Health Organization (WHO), in 2016, there were over 1.9 billion adults who were overweight, and of these, > 650 million were diagnosed with obesity [3]. Obesity may cause a variety of changes in cardiac morphology that predispose patients to ventricular dysfunction [4], and obesity is associated with an increased risk of heart failure. Previous studies have demonstrated that obesity can induce left ventricular (LV) hypertrophy, enlargement, cardiac fibrosis, and diastolic dysfunction that eventually evolves to overt heart failure [5, 6]. The subclinical impairment in LV systolic dysfunction in obese patients needs further exploration.

LV ejection fraction (LVEF) is the most commonly used echocardiographic method for assessing LV systolic function in obese patients at present. More recently, echocardiography techniques such as tissue-Doppler imaging (TDI) and speckle tracking imaging have revealed subclinical LV systolic dysfunction in subjects with obesity [7, 8], Subclinical LV systolic dysfunction means subclinical myocardial dysfunction despite a normal LVEF was detected. Myocardial work (MW), as a new technology, quantification of the pressure-strain loop (PSL) which constructed based on strain combined with noninvasively measured LV peak systolic pressure, has been used in research in recent years [9]. Previous studies have reported that MW could evaluate subclinical LV systolic dysfunction in cardiac diseases, such as CAD [10–12], hypertension [13–15], T2DM [16–18], cardiomyopathy [19, 20] and so on, and demonstrate LV systolic dysfunction. Furthermore, some scholars have reported that MW is superior to global longitudinal strain (GLS) in predicting some cardiac diseases [10]. However, MW for detecting subclinical LV systolic dysfunction in obese patients has not been reported.

The purpose of this study was to examine subclinical LV systolic dysfunction in obese patients by using the MW technique. Obese patients were further classified into mild, moderate and severe obese groups based on BMI, and the differences in subclinical LV systolic dysfunction among these subgroups were analysed.

## Subjects and methods

### Ethical statement

The research was approved by the Human Research and Ethics Committee of the Affiliated Changzhou Second People’s Hospital with Nanjing Medical University. Informed consent was obtained from each patient prior to study participation.

### Patient population and study design

From January 2019 to January 2023, we prospectively recruited the obese patients admitted to the department of weight loss metabolic surgery, the Affiliated Changzhou Second People’s Hospital with Nanjing Medical University. Patients with BMI > 30 kg/m^2^ are considered obese. A total of 589 obese patients and 100 normal subjects were included. The recruitment of these patients was consecutive. According to BMI, we divided all obese patients into three groups: class I obesity (mild) 30–35 kg/m^2^, class II obesity (moderate) 35–40 kg/m^2^ and class III obesity (severe) > 40 kg/m^2^ [1, 4]. Patients with a history of arrhythmia, congenital heart disease, myocardial infarction, cardiomyopathy, valvular disease, thyroid disease, neoplastic disease, or kidney failure were excluded from the study. One hundred normal subjects of similar age and sex were enrolled as controls.

Height, weight, heart rate and blood pressure were recorded before the echocardiography examination from all enrolled subjects, and then BMI and BSA were calculated. Laboratory tests of fasting plasma glucose (FPG), glycosylated haemoglobin (HbA1c), total cholesterol (TCH), triglyceride (TG), high density lipoprotein (HDL) and low-density lipoprotein (LDL), blood urea nitrogen (BUN), and serum creatinine (SCR) were taken when the patients were in the hospital.

### Echocardiography

Echocardiography examination was performed by using a GE Vivid E9 ultrasound diagnostic system equipped with an M5s 3.5-5 MHz transducer (GE Vingmed Ultrasound, Horten, Norway) by experienced sonographers. ECG leads were connected to each patient, and standard high frame rates (> 60/s) of apical four-chamber, two-chamber, and long-axis views of three consecutive cycles were stored for offline analysis.

Left atrial diameter (LAd), interventricular septum thickness (IVSd), LV posterior wall thickness (LVPWd), LV diameter (LVDd) in the end-diastole period and mitral annular plane systolic excursion (MAPSE) were measured by M-mode. Left atrial maximum volume (LAV) was measured in the 2D echocardiography images using Simpson′s method in the apical 4- and 2- chamber views, and then the LAV index (LVMI) was calculated. LVEF was obtained from the biplane Simpson’s method. The peak early and late diastolic mitral annular velocities (E and A, respectively) were measured by pulsed-wave Doppler, and the ratio E/A was then calculated. The peak early (e′) and late (a′) diastolic annular velocities were obtained by averaging the values at the septum and lateral positions using TDI, and E/e′ was calculated.

Two-dimensional speckle tracking echocardiography analyses.

Global MW was measured by EchoPAC software (EchoPAC Version: 203, GE Vingmed Ultrasound, Norway).

The endocardial border at the end-systolic frame in apical long-axis, four-chamber and two-chamber views was manually traced, and the software automatically created a region of interest, which included the entire myocardium. The LV myocardium was divided into 18 segments, and GLS was automatically measured by the software [21].

After the GLS was calculated, input the blood pressure into the software. PSL was constructed based on strain combined with noninvasively measured LV peak systolic pressure, and then the global MW was generated.

The global myocardial work index (GWI), global constructive work (GCW), global wasted work (GWW) and myocardial work efficiency (GWE) were automatically generated by the software.

(1) GWI: total work within the area of the LV PSL. (2) GCW: work performed by the LV contributing to LV ejection during systole. (3) GWW: work performed by the LV that does not contribute to LV ejection. (4) GWE: GCW/(GCW+GWW)*100% [15]. Then, we incorporated a new evaluation parameter, GCW/GWW, which was a more intuitive evaluation that constructs a gain/loss relationship between work and waste.

### Statistical analysis

The sample size was calculated using the PASS software (15.0.5, NCSS, LLC), Our study was powered to test the significant differences between obese patients and normal subjects. We randomly selected 20 healthy individuals and 100 obese patients and determined the sample size based on the preliminary results. Group sample sizes of 88 and 440 achieve over 95.0% power for MW to reject the null hypothesis of equal means, and with a significance level (alpha) of 0.05 using a two-sided equal-variance t-test. Therefore, we ultimately included 100 healthy individuals and 589 obese patients, providing at least 95.0% of the power for MW analysis. All data analyses were performed using SPSS 26.0 software (SPSS, Chicago, IL, USA). The normality of all values was assessed by the Shapiro‒Wilk test. Differences between the obese patients and normal controls were compared with an independent Student′s t test because the data distribution was normal. For variables with a nonnormal distribution, the nonparametric Mann‒Whitney test was used. Differences among the mild, moderate, and severe obese patients were compared with one-way analysis of variance (ANOVA) for normal distribution, while the Kruskal‒Wallis rank sum test was used for nonnormal distribution of continuous variables. Comparisons of two samples were performed using the least-significant difference (LSD) or Bonferroni test as appropriate. We define obese patients with absolute value of GLS<20% as abnormal, and obese patients with absolute value of GLS ≥ 20% as normal. The sensitivity and specificity of MW related parameters in evaluating subclinical LV systolic dysfunction in obese patients were determined from receiver operating characteristic (ROC) curve analysis by MedCalc Statistical Software, v.19.6.4 (Ostend, Belgium). Youden′s index was selected as the cut-off point that can give the best composite of specificity and sensitivity. The difference in the area under the ROC curve (AUC) between the two different methods and the combined two methods were tested with the DeLong method. Correlations among GWI were tested using Pearson tests. In univariable linear regression, the outcomes of the factors with *P* < 0.05 were incorporated into the multivariable linear regression analysis models to detect the independent predictors of abnormal LV myocardial function in obese patients. Variables are depicted as the mean ± standard deviation, median (interquartile range), or percentages as appropriate. A *P* value < 0.05 was considered significant in all tests.

### Reproducibility and repeatability

Interobserver and interobserver variabilities in GWI, GCW, GWW, GWE and GCW/GWW were determined by repeating measurements in 20 randomly selected patients among obese patients and normal subjects. For the second interobserver measurements, the observer was “blinded” to the results of the initial measurements.

## Results

### Patient characteristics and laboratory examination results between normal controls and obese patients are shown in Table 1

**Table 1:** Clinical parameters of normal controls and obese patients

Clinical parameters	Normal controls (n = 100)	Obesity (n = 589)	*P* value
Age, year	32.28 ± 7.05	31.54 ± 7.60	0.361
Male, n (%)	35(35)	153(26)	0.061
Height, cm	165.64 ± 8.37	167.13 ± 8.01	0.089
Weight, kg	62.94 ± 11.52	105.4 ± 20.71	< 0.001
BMI, kg/m^2^	22.80 ± 2.79	37.53 ± 5.40	< 0.001
BSA, m^2^	1.66 ± 0.19	2.22 ± 0.30	< 0.001
SBP, mmHg	120.64 ± 10.96	133.48 ± 16.87	< 0.001
DBP, mmHg	76.07 ± 7.91	87.18 ± 12.06	< 0.001
h, bpm	74.00(73.13,77.49)	80.00(80.17,82.29)	< 0.001
FPG, mmol/L	4.95(4.68,5.08)	5.69(6.34,6.74)	< 0.001
HbA1c, %	5.34 ± 0.40	6.24 ± 1.33	0.002
TC, mmol/L	4.40 ± 0.85	4.84 ± 0.96	0.013
TG, mmol/L	1.06(0.97,1.30)	1.66(1.97,2.31)	0.012
HDL-C, mmol/L	1.28 ± 0.23	1.13 ± 0.48	0.003
LDL-C, mmol/L	2.62 ± 0.85	3.16 ± 0.78	< 0.001
BUN, mmol/L	4.65(3.89,4.89)	4.60(4.59,4.77)	0.311
SCR, μmol/L	59.00(55.30,68.01)	58.00(58.86,63.23)	0.565
Complications, %			
Hypertension	0	120(20)	
T2DM	0	242(41)	

BMI: body mass index, BSA: body surface area, SBP: systolic blood pressure, DBP: diastolic blood pressure. HR: heart rate, FPG: fasting plasma glucose, HbA1c: glycated haemoglobin, TC: total cholesterol, TG: triglyceride, HDL-C: high-density lipoprotein, LDL-C: low-density lipoprotein, BUN: blood urea nitrogen, SCR: serum creatinine

There were significant differences in weight, BMI, BSA, SBP, DBP and HR between normal controls and obese patients (*P* < 0.001). The values of weight, BMI, BSA, SBP, DBP and HR in obese patients were significantly larger than those in normal controls. There were no significant differences in age or sex (*P* > 0.05).

The values of HbA1c, TC, TG, LDL-C and FPG in obese patients were significantly larger than those in normal controls (*P* < 0.05), and there were no significant differences in BUN and SCR (*P* > 0.05).

### Echocardiographic parameters and GLS between normal controls and obese patients (Table 2)

**Table 2:** Echocardiographic parameters between normal controls and obese patients

Echocardiographic parameters	Normal controls (n = 100)	Obesity (n = 589)	*P* value
LA diameter, mm	32.68 ± 2.88	37.39 ± 3.78	< 0.001
LAV index, ml/m^2^	27.37 ± 6.71	26.61 ± 6.53	0.283
IVS diameter, mm	8.82 ± 0.93	9.97 ± 1.09	< 0.001
LVPW diameter, mm	8.75 ± 2.41	9.90 ± 2.54	< 0.001
LV diameter, mm	45.80 ± 3.08	48.86 ± 4.66	< 0.001
LVEDV, ml	78.66 ± 17.59	89.10 ± 26.88	< 0.001
LVESV, ml	28.57 ± 7.93	34.18 ± 12.70	< 0.001
LVEF, %	63.88 ± 3.86	62.05 ± 4.06	< 0.001
MAPSE, mm	14.56 ± 1.52	14.89 ± 1.73	0.073
E, m/s	0.88 ± 0.15	0.81 ± 0.16	< 0.001
A, m/s	0.62 ± 0.15	0.71 ± 0.17	< 0.001
E/A	1.44(1.40,1.54)	1.16(1.17,1.24)	< 0.001
e′, m/s	0.14 ± 0.02	0.11 ± 0.02	< 0.001
E/e′	6.36(6.22,6.67)	7.30(7.34,7.64)	< 0.001
GLS, %	-21.91 ± 1.89	-19.28 ± 2.79	< 0.001

LAd: left atrial diameter, LAV index: left atrial volume index, IVSd: interventricular septal wall thickness in the end-diastolic period, LVPWd: left ventricular posterior wall thickness in the end-diastolic period, LVDd: left ventricular diameter in the end-diastolic period, LVEDV: left ventricular end-diastolic volume, LVESV: left ventricular end-systolic volume, LVEF: left ventricular ejection fraction, MAPSE: mitral annular plane systolic excursion, E: peak velocity during early diastole of the anterior mitral leaflet, A: peak velocity during late diastole of the anterior mitral leaflet, e′: peak early diastolic annular velocities using TDI by averaging the values at the septum and lateral positions. GLS: global longitudinal strain

There were significant differences in LAD, IVSd, LVPWd, LVd, LVEDV, LVESV, LVEF, E, A, E/A, e′, and E/e′ between normal controls and obese patients (*P* < 0.001). The values of IVSd, LVPWd, LVd, LVEDV, LVESV, A, and E/e′ in obese patients were significantly larger than those in normal controls; however, the values of LVEF, E, E/A, and e′ were significantly lower than those in normal controls. There were no significant differences in the LAV index or MAPSE among normal controls, T2DM patients and T2DM patients with HT (*P* > 0.05).

The absolute value of GLS in obese patients was significantly lower than that in normal controls (*P* < 0.001).

### Global MW between normal controls and obese patients (Table 3; Fig. 1)

**Table 3:** Global myocardial work index between normal controls and obese patients

Global MW	Normal controls	Obesity	*P* value	*P*_trend_	Obesity		*P* value
	(n = 100)	(n = 589)			normal GLS (n = 262)	abnormal GLS (n = 327)	
GWI, mmHg%	2183.91 ± 309.13	2095.59 ± 375.66	0.012	0.026	2309.33 ± 332.81^*^	1924.34 ± 315.78^*#^	< 0.001
GCW, mmHg%	2623.08 ± 354.49	2515.34 ± 401.48	0.012	0.012	2750.15 ± 337.48^*^	2327.21 ± 346.02^*#^	< 0.001
GWW, mmHg%	62.00(65.54,85.00)	89.00(103.57,117.69)	< 0.001	< 0.001	82.00(86.22,101.81) ^*^	96.00(113.05,134.85) ^*#^	< 0.001
GWE, %	97.00(96.43,96.99)	96.00(94.94,95.41)	< 0.001	< 0.001	97.00(95.97,96.40) ^*^	95.00(94.01,94.73) ^*#^	< 0.001
GCW/GWW	41.37(41.97,52.37)	28.03(31.80,35.75)	< 0.001	< 0.001	33.89(37.91,44.44) ^*^	23.74(25.62,30.07) ^*#^	< 0.001

GWI: global myocardial work index, GCW: global constructive work, GWW: global wasted work, GWE: myocardial work efficiency, GLS: global longitudinal strain

^*^Significantly different (*P* < 0.05) when obesity with normal GLS or obesity with abnormal GLS were compared with normal controls

^#^ Significantly different (*P* < 0.05) when obesity with abnormal GLS was compared with normal GLS.

**Fig. 1:**
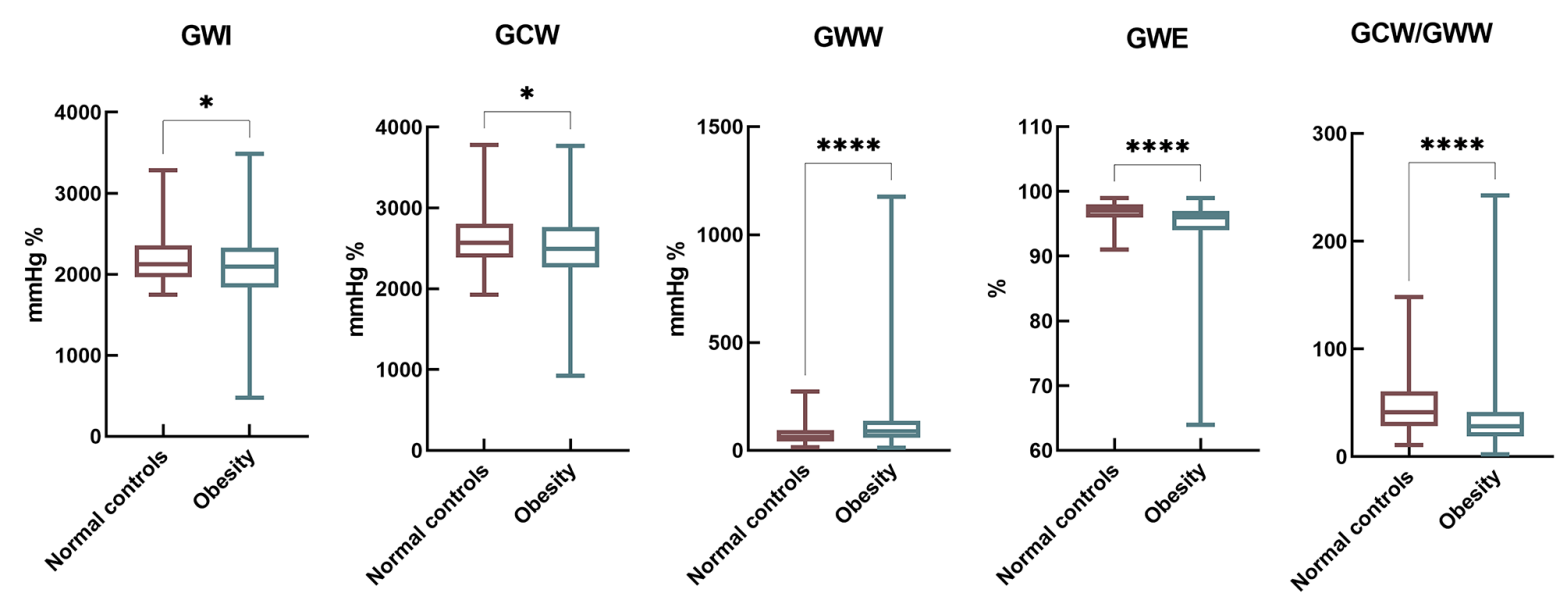
Global myocardial work between normal controls and obese patients

There were significant differences in GWI, GCW, GWW, GWE and GCW/GWW between normal controls and obese patients (*P* < 0.005). The values of GWI, GCW, GWE and GCW/GWW in obese patients were significantly lower than those in normal controls, while GWW was significantly larger than that in normal controls. The GWI, GCW, and GWW of obese patients with normal GLS were significantly increased compared to the normal controls (*P* < 0.05), but GWE and GCW/GWW were significantly reduced compared to the normal controls (*P* < 0.05). The GWI, GCW, GWE, and GCW/GWW of obese patients with abnormal GLS were significantly reduced compared to the normal controls and obese patients with normal GLS (*P* < 0.05), while GWW was significantly increased compared to the normal controls and obese patients with normal GLS (*P* < 0.05).

### Subgroup analysis of global MW in obese patients (Table 4; Fig. 2)

**Table 4:** Global myocardial work index among patients with mild, moderate and severe obesity

Global MW	Mild obesity (n = 224)	Moderate obesity (n = 209)	Severe obesity (n = 156)	*P* value	P _trend_
GWI, mmHg%	2136.08 ± 369.47	2121.23 ± 355.06	2003.12 ± 397.37^*#^	0.003	0.001
GCW, mmHg%	2565.28 ± 377.60	2523.51 ± 407.64	2432.69 ± 415.65^*#^	0.006	0.002
GWW, mmHg%	79.50(91.52,119.08)	87.00(95.73,115.29)	104.50(113.00,137.31) ^*#^	< 0.001	0.029
GWE, %	96.00(95.21,96.03)	96.00(95.06,95.71)	95.00(93.79,94.73) ^*#^	< 0.001	< 0.001
GCW/GWW	32.40(33.63,39.56)	28.80(30.59,36.15)	22.61(25.39,35.13) ^*#^	< 0.001	0.013

*Significantly different (*P* < 0.05) when severe obesity was compared with mild obesity

# Significantly different (*P* < 0.05) when severe obesity was compared with moderate obesity

**Fig. 2:**
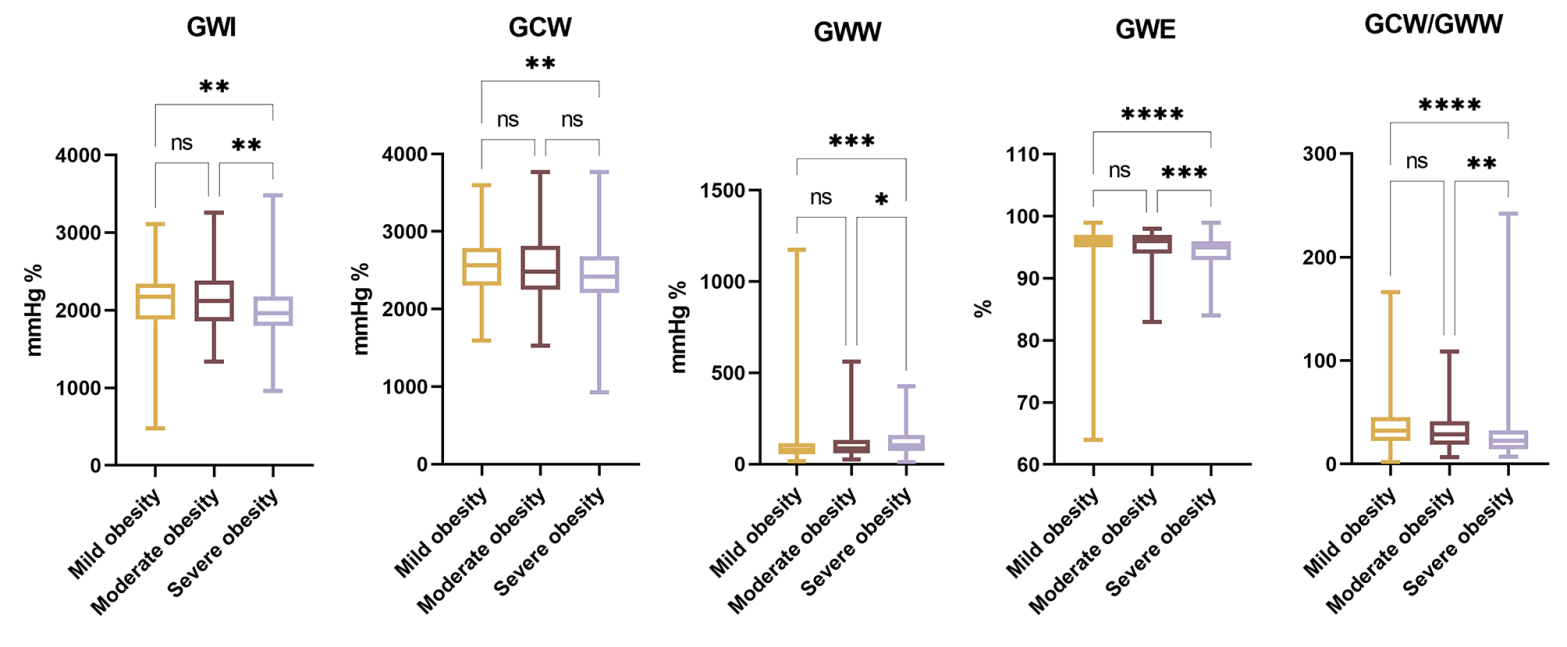
Global myocardial work index among Mild, Moderate and Severe obese patients

There were significant differences in GWI, GCW, GWW, GWE and GCW/GWW among mild, moderate and severe obese patients (*P* < 0.001). Trend analysis showed that the values of GWI, GCW, GWE and GCW/GWW in severe obese patients were lower than those in moderate obese patients and lower than those in mild obese patients (*P* < 0.01), while GWW in severe obese patients was larger than that in moderate obese patients and larger than that in mild obese patients (*P* < 0.05).

### Univariable and multivariate regression for GWI and GCW in obese patients (Tables 5 and 6)

**Table 5:** Relationship between GWI and different clinical and echocardiographic parameters in obese patients

GWI	Univariable analysis					Multivariable analysis			
	*B*	*r*	95%CI	*p*		*B*	*β*	95%CI	*p*
sex	158.41	0.19	90.22 to 226.60	< 0.001		128.10	0.15	69.26 to 186.93	< 0.001
Age, year	8.70	0.18	4.76 to 12.64	< 0.001		-0.28	-0.01	-3.62 to 3.07	0.871
BMI, kg/m^2^	-13.60	-0.20	-19.13 to -8.07	< 0.001		-12.11	-0.18	-16.94 to -7.28	< 0.001
HbA1c, %	-20.26	-0.07	-43.33 to 2.80	0.085		-23.72	-0.08	-42.61 to -4.82	0.014
TG, mmol/L	-12.09	-0.07	-26.56 to 2.38	0.101		N/A	N/A	N/A	N/A
LDL-C, mmol/L	4.31	0.01	-35.07 to 43.68	0.830		N/A	N/A	N/A	N/A
FPG, mmol/L	-6.73	-0.04	-19.30 to 5.83	0.293		N/A	N/A	N/A	N/A
SBP, mmHg	8.21	0.37	6.53 to 9.89	< 0.001		11.02	0.50	9.49 to 12.55	< 0.001
LVEF, %	37.20	0.40	30.32 to 44.08	< 0.001		35.50	0.39	29.33 to 41.67	< 0.001

**Table 6:** Relationship between GCW and different clinical and echocardiographic parameters in obese patients

GCW	Univariable analysis					Multivariable analysis			
	*B*	*r*	95%CI	*p*		*B*	*β*	95%CI	*p*
sex	156.50	0.17	83.43 to 229.56	< 0.001		153.27	0.17	93.93 to 212.62	< 0.001
Age, year	8.16	0.15	3.93 to 12.39	< 0.001		-2.83	-0.05	-6.19 to 0.52	0.098
BMI, kg/m^2^	-12.75	-0.17	-18.68 to -6.81	< 0.001		-13.86	-0.19	-18.67 to -9.05	< 0.001
HbA1c, %	-2.91	-0.01	-27.77 to 21.94	0.818		N/A	N/A	N/A	N/A
TG, mmol/L	-10.35	-0.05	-25.79 to 5.10	0.189		N/A	N/A	N/A	N/A
LDL-C, mmol/L	11.90	0.02	-30.08 to 53.87	0.578		N/A	N/A	N/A	N/A
FPG, mmol/L	5.29	0.03	-8.11 to 18.69	0.439		N/A	N/A	N/A	N/A
SBP, mmHg	10.45	0.44	8.72 to 12.19	< 0.001		13.44	0.57	11.91 to 14.97	< 0.001
LVEF, %	39.00	0.39	31.63 to 46.38	< 0.001		39.07	0.40	32.84 to 45.30	< 0.001

Univariable linear regression analysis showed that GWI and GCW were associated with sex, age, BMI, SBP and LVEF in obese patients, so these variables were incorporated into the multivariate linear regression model of GWI and GCW by means of stepwise selection based on the univariate linear regression analysis results.

Female sex, BMI and SBP were independent influencing factors of impaired GWI (*β* = 0.15, *P* < 0.001) (*β*=-0.18, *P* < 0.001) (*β* = 0.50, *P* < 0.001) and GCW (*β* = 0.17, *P* < 0.001) (*β*=-0.19, *P* < 0.001) (*β* = 0.57, *P* < 0.001).

### ROC analysis to confirm the diagnostic value of LV dysfunction by global MW (Table 7; Fig. 3)

**Table 7:** Receiver operating characteristic curve analysis for detecting subclinical LV myocardial systolic dysfunction in obese patients

ROC analysis	GWI	GCW	GWW	GWE	GCW/GWW	Combined
Sensitivity, %	80.73	67.89	43.12	39.76	65.75	80.43
Specificity, %	69.47	84.35	72.90	88.17	60.69	80.92
Youden index	0.50	0.52	0.16	0.28	0.26	0.61
AUC (95%CI)	0.809 (0.774 to 0.840)	0.820 (0.786 to 0.850)	0.603 (0.562 to 0.643)	0.696 (0.657 to 0.733)	0.684 (0.645 to 0.721)	0.872 (0.842 to 0.897)
Associated criterion	2163.00	2442.00	109.00	94.00	29.78	0.55
*P* value	< 0.001	< 0.001	< 0.001	< 0.001	< 0.001	

*P* values indicate the combined evaluation compared with a single global MW index

**Fig. 3:**
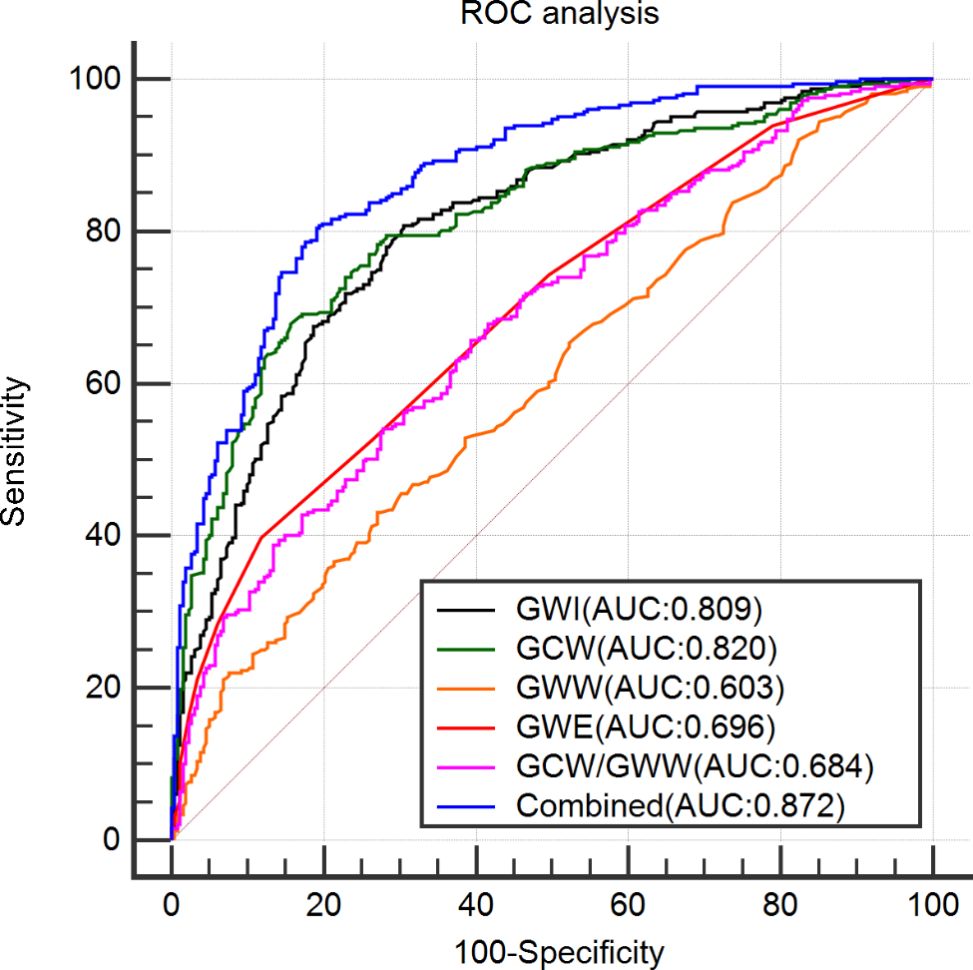
ROC analysis was performed to determine the performance of MW to detect subclinical LV systolic dysfunction in obese patients

The AUC of GWI was 0.809 (0.774 to 0.840), and the cut-off value was 2163.00mmHg%, with a sensitivity of 80.73% and specificity of 69.47%. The AUC of GCW was 0.820 (0.786 to 0.850), and the cut-off value was 2442.00 mmHg%, with a sensitivity of 67.89% and specificity of 84.35%. The AUC of GWW was 0.603 (0.562 to 0.643), and the cut-off value was 109.00 mmHg%, with a sensitivity of 43.12% and specificity of 72.90%. The AUC of GWE was 0.696 (0.657 to 0.733), and the cut-off value was 94.00%, with a sensitivity of 39.76% and specificity of 88.17%. The AUC of GCW/GWW was 0.684 (0.645 to 0.721), and the cut-off value was 29.78, with a sensitivity of 65.75% and specificity of 60.69%.

The AUC of the combination of GWI, GCW, GWW, and GWE was 0.872 (0.842 to 0.897), and the cut-off value was 0.55, with a sensitivity of 80.43% and specificity of 80.92%. The AUC value was significantly higher than the AUCs of the individual indices (*P* < 0.001).

### Intraobserver and interobserver variability are presented in Table 8

**Table 8:** ICCs for intra- and interobserver variability for MW parameters

Variable	Interobserver variability		Intraobserver variability	
	ICC	95% CI	ICC	95% CI
GWI (mmHg %)	0.949	0.872–0.980	0.959	0.895–0.984
GCW (mmHg %)	0.959	0.896–0.984	0.966	0.913–0.986
GWW (mmHg %)	0.975	0.938–0.990	0.980	0.949–0.992
GWE (%)	0.967	0.916–0.987	0.966	0.915–0.987
GCW/GWW	0.948	0.869–0.979	0.954	0.883–0.982

Intraobserver and interobserver variabilities were calculated by the intraclass correlation coefficient (ICC). All global MW parameters exhibited excellent intra- and interobserver correlations with ICC values > 0.94.

## Discussion

The main findings of the study were that subclinical LV systolic dysfunction was detected by the novel technique global MW in obese patients. Subgroup analysis showed that patients with severe obesity had the most severe subclinical LV systolic dysfunction, were followed by moderate obesity and mild obesity.

Obesity has a significant impact on the cardiac system; not only is obesity closely intertwined with a greater prevalence of coronary artery disease, hypertension, T2DM and OSAS, but obesity alone also impacts myocardial structure and pump performance [5]. Echocardiography is an important tool to provide an estimate of LV function. LVEF, as the most relevant parameter, is not sensitive enough to detect subclinical myocardial damage and is therefore not suitable for detecting subclinical LV systolic dysfunction [22]. GLS is considered an accurate method for evaluating subclinical myocardial function and is widely used in scientific research and clinical practice. Kalisz, K et al. [23]. used cardiac MRI to measure LVGLS to demonstrate ventricular function in obese subjects in the absence of clinically apparent cardiovascular disease and found a significant decrease in LVGLS in obese versus nonobese subjects without differences in ejection fraction and indexed LV mass and in the absence of other comorbidities. Kibar AE et al. [24] used GLS calculated by STE to assess the effect of childhood obesity on LV function and found that childhood obesity, in the absence of hypertension, is associated with an altered longitudinal LV function by STE. Blomstrand P et al. [25] found that overweight and obesity impaired LVEF and GLS in both patients with T2DM and nondiabetic persons. Although impaired GLS in obese patients can indicate subclinical LV myocardial systolic dysfunction, significant differences in MW related parameters were observed in obese patients with normal GLS compared to the normal controls in the present study. GWI and GCW showed a significant increase compared to the normal controls, which may be due to increase of SBP in some obese patients. Due to the better consideration of afterload compared to GLS in MW, GWI and GCW showed an increase, but this does not seem to suggest that they are in a healthy state. Moreover, obese patients with normal GLS showed a significant increase in GWW and a significant decrease in GWE and GCW/GWW compared to the normal controls, which seems to provide more reference value for subclinical LV myocardial systolic dysfunction in obese patients with normal GLS.

In this research, we innovatively used a novel parameter, MW, to evaluate subclinical LV systolic dysfunction in obese patients. From our results, the standard echocardiographic evaluation of obese patients had higher morphological and functional echocardiographic abnormalities, such as larger LA and LV diameters, LV volumes, thickened LV wall, and reduced LVEF, although they were in the normal range, and the results are consistent with previous research [26]. LV dilatation and hypertrophy as a response to sustained pressure overload and extended wall stress decreased LV longitudinal function. The results are consistent with the GLS we measured. LV GLS in obese patients is significantly decreased compared to that in normal controls.

From global MW analysis, we found that GWI, GCW, GWE and GCW/GWW in obese patients were significantly decreased, while GWW was significantly increased compared with normal controls. LV enlargement and LV hypertrophy can damage the subendocardial myocardial fibres, which are responsible for myocardial function. Animal experiments have shown that the levels of TGF-β1 and leptin were overtly increased in cardiac tissue from obese rabbits compared with lean rabbits [27, 28]. TGF- β1 is closely related to myocardial fibrosis, and leptin coincides with cardiac hypertrophy through binding of leptin to the short form leptin receptor in rat hearts. Cardiac hypertrophy and fibrosis are strongly associated with obesity, and metabolic dysfunction can induce LV systolic dysfunction and may contribute to the increased incidence of heart failure and sudden cardiac death in obese subjects [29]. In addition, elevated LV wall stress in obesity evokes the increases in myocardial oxygen consumption. The increases in substrate supply in the hearts of obese patients trigger an increase in fatty acid oxidation in conjunction with suppressed glucose oxidation, resulting in impaired cardiac efficiency and cardiac systolic dysfunction [5, 30].

Subgroup analysis showed that the presence of subclinical LV systolic dysfunction in all classes of obesity worsened with increasing BMI but may not be characterized by abnormal LV ejection phase indices. From the results, we know that elevated BMI in obese patients results in an increased risk of subclinical LV systolic dysfunction.

Univariable and multivariable regression analyses revealed that female sex, BMI and SBP were independent influencing factors of impaired GWI. Female sex hormones such as oestrogen can interact with certain risk factors to precipitate myopathic changes in the heart [31], and this view may verify this result. Controlling BMI in obese patients may reduce the impairment to the LV myocardial systolic function.

ROC analysis showed that the combination of GWI, GCW, GWW, and GWE has good sensitivity and specificity (> 80%) in evaluating subclinical LV myocardial systolic dysfunction in obese patients.

The intraobserver and interobserver variabilities were low in the study, which supports the view that the measurements are valid.

## Conclusions

In this study, we conclude that subclinical LV systolic dysfunction was detected by the novel global MW technique in obese patients. Elevated BMI in obese patients result in an increased risk of subclinical LV systolic dysfunction, although the LVEF is normal. Controlling BMI in obese patients may reduce the impairment to the LV myocardial systolic function. Global MW is a novel and reproducible technique that can be well applied in the clinical evaluation of subclinical LV systolic dysfunction.

### Limitations

Our study had some limitations. For the first, the study is a single centre research. Second, there were fewer men than women in the patient population. Men with obesity are more likely to be affected by concentric cardiac hypertrophy than eccentric hypertrophy while women with obesity experience both types of hypertrophies [32]. Third, since many obese patients exhibit insulin resistance, impaired glucose tolerance, or overt diabetes mellitus, it is often difficult to determine whether LV systolic dysfunction in obese patients is independently attributable to obesity.

## Data Availability

The datasets used and analyzed during the current study are available from the corresponding author on reasonable request.

## Abbreviations

A: Peak velocity during late diastole of the anterior mitral leaflet
BMI: Body mass index
BSA: Body surface area
BUN: Blood urea nitrogen
DBP: Diastolic blood pressure
e′: Peak early diastolic annular velocities using TDI by averaging the values at the septum and lateral positions
E: Peak velocity during early diastole of the anterior mitral leaflet
FPG: Fasting plasma glucose
GCW: Global constructive work
GLS: Global longitudinal strain
GWE: Myocardial work efficiency
GWI: Global myocardial work index
GWW: Global wasted work
HbA1c: Glycated haemoglobin
HDL-C: High-density lipoprotein
HR: Heart rate
IVSd: Interventricular septal wall thickness in the end-diastolic period
LAd: Left atrial diameter
LAV index: Left atrial volume index
LDL-C: Low-density lipoprotein
LVDd: Left ventricular diameter in the end-diastolic period
LVEDV: Left ventricular end-diastolic volume
LVEF: Left ventricular ejection fraction
LVESV: Left ventricular end-systolic volume
LVPWd: Left ventricular posterior wall thickness in the end-diastolic period
MAPSE: Mitral annular plane systolic excursion
SBP: Systolic blood pressure
SCR: Serum creatinine
TC: Total cholesterol
TG: Triglyceride
